# Bis(9-allyl-6-carb­oxy-9*H*-carbazole-3-carboxyl­ato-κ^2^ *O* ^3^,*O* ^3′^)diaqua­zinc

**DOI:** 10.1107/S1600536812045357

**Published:** 2012-11-10

**Authors:** Dailin Li

**Affiliations:** a Environmental Engineering Department, Xiamen University of Technology, Xiamen 361024, People’s Republic of China

## Abstract

In the title compound, [Zn(C_17_H_12_NO_4_)_2_(H_2_O)_2_], the Zn^II^ atom is located on a twofold rotation axis and is six-coordinated by four carboxyl­ate O atoms from two chelating 9-allyl-6-carb­oxy-9*H*-carbazole-3-carboxyl­ate ligands and two O atoms from two water mol­ecules. In the crystal, O—H⋯O hydrogen bonds link the mol­ecules into a layer structure parallel to (-101).

## Related literature

For the design and properties of complexes with supra­molecular metal-organic framework structures, see: Li *et al.* (2011 ▶); Yang *et al.* (2007 ▶). For related structures, see: Wang *et al.* (2010 ▶).
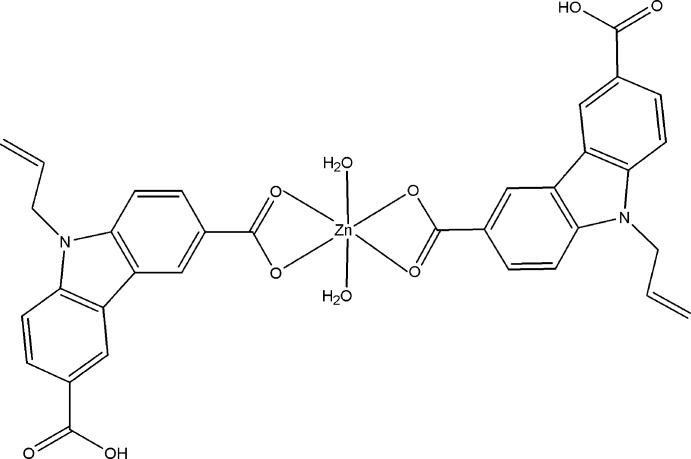


## Experimental

### 

#### Crystal data

[Zn(C_17_H_12_NO_4_)_2_(H_2_O)_2_]
*M* *_r_* = 689.95
Monoclinic, 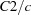

*a* = 30.8562 (18) Å
*b* = 5.0491 (3) Å
*c* = 21.8915 (13) Å
β = 119.403 (1)°
*V* = 2971.3 (3) Å^3^
*Z* = 4
Mo *K*α radiation
μ = 0.89 mm^−1^
*T* = 173 K
0.22 × 0.16 × 0.14 mm

#### Data collection

Bruker APEXII CCD diffractometer
Absorption correction: multi-scan (*SADABS*; Bruker, 2001 ▶) *T*_min_ = 0.828, *T*_max_ = 0.885
7758 measured reflections
2942 independent reflections
2390 reflections with *I* > 2σ(*I*)
*R*_int_ = 0.033

#### Refinement

*R*[*F* ^2^ > 2σ(*F* ^2^)] = 0.036
*wR*(*F* ^2^) = 0.100
*S* = 1.02
2942 reflections
213 parameters
H-atom parameters constrained
Δρ_max_ = 0.27 e Å^−3^
Δρ_min_ = −0.26 e Å^−3^

### 

Data collection: *APEX2* (Bruker, 2007 ▶); cell refinement: *SAINT* (Bruker, 2007 ▶); data reduction: *SAINT*; program(s) used to solve structure: *SHELXTL* (Sheldrick, 2008 ▶); program(s) used to refine structure: *SHELXTL*; molecular graphics: *XP* in *SHELXTL* and *Mercury* (Macrae *et al.*, 2006 ▶); software used to prepare material for publication: *SHELXTL*.

## Supplementary Material

Crystal structure: contains datablock(s) global, I. DOI: 10.1107/S1600536812045357/vn2058sup1.cif

Structure factors: contains datablock(s) I. DOI: 10.1107/S1600536812045357/vn2058Isup2.hkl

Additional supplementary materials:  crystallographic information; 3D view; checkCIF report

## Figures and Tables

**Table 1:** Selected bond lengths (Å)

Zn1—O1	2.4040 (15)
Zn1—O2	2.0392 (15)
Zn1—O1*W*	1.9824 (16)

**Table 2:** Hydrogen-bond geometry (Å, °)

*D*—H⋯*A*	*D*—H	H⋯*A*	*D*⋯*A*	*D*—H⋯*A*
O1*W*—H1*A*⋯O4^i^	0.84	1.81	2.654 (2)	177
O1*W*—H1*B*⋯O2^ii^	0.85	1.88	2.728 (2)	170
O3—H3*A*⋯O1^i^	0.87	1.77	2.634 (2)	173

Symmetry codes: (i) 
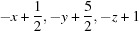
; (ii) 
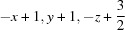
.

**Fig. 1.**
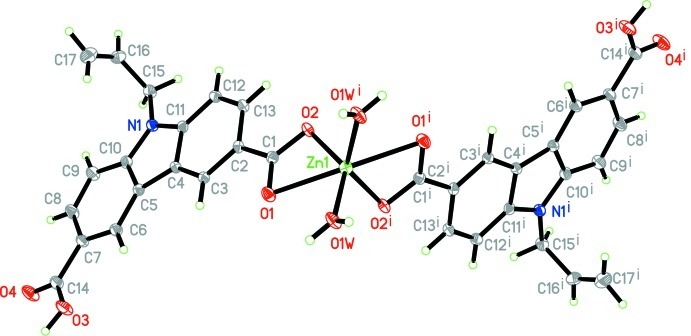
Molecular structure of the title compound. Displacement ellipsoids are drawn at the 30% probability level. [Symmetry code: (i) 1 - x, y, 3/2 - z.]

**Fig. 2.**
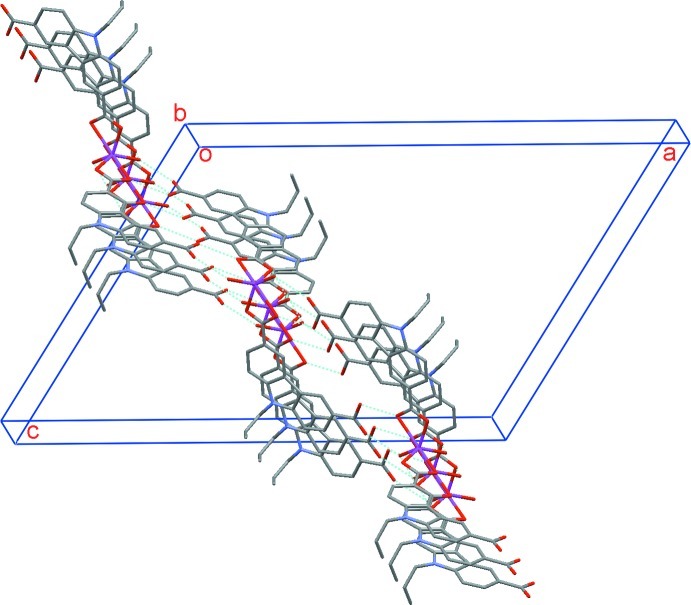
View of the layer structure in the title compound, built by hydrogen bonds (dashed lines).

## supplementary crystallographic information

### Comment

Crystal engineering based on metal-organic frameworks (MOFs) continues to attract considerable interest not only because of their intriguing variety of architectures but also for their potential functional properties, such as magnetism, nonlinear optics, hydrogen storage and catalysis (Li *et al.*, 2011; Yang *et al.*, 2007). Multifunctional ligands can link metal ions into one-, two- or three-dimensional structures. In order to extend the investigations in this field, we designed and synthesized the title zinc(II) compound and report here its structure.

The asymmetric unit of the title complex (Fig. 1) contains a Zn^II^ atom, one 9-allyl-9*H*-carbazole-6-carboxy-3-carboxylate ligand and one coordinated water molecule. The Zn^II^ atom, lying on a twofold rotation axis, is six-coordinated by four O atoms from two carboxylate ligands and two water molecules in an irregular geometry. The bond distances (Table 1) and angles are normal (Wang *et al.*, 2010). In the crystal structure, O—H···O hydrogen bonds (Table 2) link the complex molecules into a layer structure parellel to (-1 0 1) (Fig. 2).

### Experimental

The synthesis was performed under hydrothermal conditions. A mixture of 9-allyl-9*H*-carbazole-3,6-dicarboxylic acid (0.2 mmol, 0.062 g), Zn(NO_3_)_2_.6H_2_O (0.1 mmol, 0.030 g) and H_2_O (15 ml) in a 25 ml stainless steel reactor with a Teflon liner was heated from 293 to 433 K in 2 h and maintained at 433 K for 72 h. Hereafter the mixture was cooled to 298 K, and colorless crystals of the title compound were obtained (yield: 59%).

### Refinement

All H atoms on C atoms were positioned geometrically and refined as riding atoms, with C—H = 0.95 and 0.99 Å and with *U*_iso_(H) = 1.2*U*_eq_(C). H atoms bonded to O atoms were located in a difference Fourier map and refined as riding, with *U*_iso_(H) = 1.5*U*_eq_(O). Reflection (2 0 0) was affected by the beamstop shadow and excluded from the refinement by an *OMIT* instruction.

### Crystal data

**Table:**

[Zn(C_17_H_12_NO_4_)_2_(H_2_O)_2_]	*F*(000) = 1424
*M**_r_* = 689.95	*D*_x_ = 1.542 Mg m^−^^3^
Monoclinic, *C*2/*c*	Mo *K*α radiation, λ = 0.71073 Å
Hall symbol: -C 2yc	Cell parameters from 3306 reflections
*a* = 30.8562 (18) Å	θ = 2.7–26.1°
*b* = 5.0491 (3) Å	µ = 0.89 mm^−^^1^
*c* = 21.8915 (13) Å	*T* = 173 K
β = 119.403 (1)°	Block, colourless
*V* = 2971.3 (3) Å^3^	0.22 × 0.16 × 0.14 mm
*Z* = 4	

### Data collection

**Table:**

Bruker APEXII CCD diffractometer	2942 independent reflections
Radiation source: fine-focus sealed tube	2390 reflections with *I* > 2σ(*I*)
Graphite monochromator	*R*_int_ = 0.033
φ and ω scans	θ_max_ = 26.1°, θ_min_ = 1.9°
Absorption correction: multi-scan (*SADABS*; Bruker, 2001)	*h* = −38→37
*T*_min_ = 0.828, *T*_max_ = 0.885	*k* = −4→6
7758 measured reflections	*l* = −27→20

### Refinement

**Table:**

Refinement on *F*^2^	Primary atom site location: structure-invariant direct methods
Least-squares matrix: full	Secondary atom site location: difference Fourier map
*R*[*F*^2^ > 2σ(*F*^2^)] = 0.036	Hydrogen site location: inferred from neighbouring sites
*wR*(*F*^2^) = 0.100	H-atom parameters constrained
*S* = 1.02	*w* = 1/[σ^2^(*F*_o_^2^) + (0.058*P*)^2^] where *P* = (*F*_o_^2^ + 2*F*_c_^2^)/3
2942 reflections	(Δ/σ)_max_ = 0.001
213 parameters	Δρ_max_ = 0.27 e Å^−^^3^
0 restraints	Δρ_min_ = −0.26 e Å^−^^3^

### Special details

**Table:**

Geometry. All e.s.d.'s (except the e.s.d. in the dihedral angle between two l.s. planes) are estimated using the full covariance matrix. The cell e.s.d.'s are taken into account individually in the estimation of e.s.d.'s in distances, angles and torsion angles; correlations between e.s.d.'s in cell parameters are only used when they are defined by crystal symmetry. An approximate (isotropic) treatment of cell e.s.d.'s is used for estimating e.s.d.'s involving l.s. planes.
Refinement. Refinement of *F*^2^ against ALL reflections. The weighted *R*-factor *wR* and goodness of fit *S* are based on *F*^2^, conventional *R*-factors *R* are based on *F*, with *F* set to zero for negative *F*^2^. The threshold expression of *F*^2^ > σ(*F*^2^) is used only for calculating *R*-factors(gt) *etc*. and is not relevant to the choice of reflections for refinement. *R*-factors based on *F*^2^ are statistically about twice as large as those based on *F*, and *R*- factors based on ALL data will be even larger.

### Fractional atomic coordinates and isotropic or equivalent isotropic displacement parameters (Å^2^)

**Table:**

	*x*	*y*	*z*	*U*_iso_*/*U*_eq_	
Zn1	0.5000	1.62460 (7)	0.7500	0.02411 (14)	
C1	0.45216 (8)	1.3295 (4)	0.64103 (11)	0.0209 (5)	
C2	0.43305 (8)	1.1274 (4)	0.58455 (11)	0.0208 (5)	
C3	0.38251 (8)	1.0739 (4)	0.54621 (11)	0.0216 (5)	
H3	0.3596	1.1705	0.5547	0.026*	
C4	0.36580 (8)	0.8774 (4)	0.49528 (11)	0.0214 (5)	
C5	0.31714 (8)	0.7713 (5)	0.44657 (11)	0.0225 (5)	
C6	0.26914 (9)	0.8305 (5)	0.43223 (12)	0.0254 (5)	
H6	0.2633	0.9672	0.4571	0.030*	
C7	0.22985 (8)	0.6862 (5)	0.38084 (12)	0.0266 (5)	
C8	0.23887 (9)	0.4838 (5)	0.34407 (13)	0.0330 (6)	
H8	0.2115	0.3861	0.3095	0.040*	
C9	0.28585 (9)	0.4234 (5)	0.35656 (13)	0.0316 (6)	
H9	0.2915	0.2885	0.3310	0.038*	
C10	0.32518 (9)	0.5698 (5)	0.40878 (11)	0.0251 (5)	
C11	0.40064 (8)	0.7341 (5)	0.48402 (11)	0.0218 (5)	
C12	0.45129 (8)	0.7873 (5)	0.52155 (11)	0.0255 (5)	
H12	0.4743	0.6911	0.5132	0.031*	
C13	0.46686 (8)	0.9849 (5)	0.57137 (11)	0.0245 (5)	
H13	0.5013	1.0260	0.5975	0.029*	
C14	0.17785 (9)	0.7384 (5)	0.36266 (12)	0.0297 (6)	
C15	0.39846 (9)	0.3622 (5)	0.40603 (12)	0.0281 (5)	
H15A	0.3796	0.1938	0.3946	0.034*	
H15B	0.4327	0.3252	0.4441	0.034*	
C16	0.40095 (10)	0.4511 (6)	0.34362 (13)	0.0386 (7)	
H16	0.4190	0.3423	0.3285	0.046*	
C17	0.38062 (11)	0.6661 (7)	0.30711 (15)	0.0517 (8)	
H17A	0.3622	0.7812	0.3202	0.062*	
H17B	0.3843	0.7069	0.2675	0.062*	
N1	0.37548 (7)	0.5493 (4)	0.43157 (9)	0.0238 (4)	
O1	0.42355 (6)	1.4699 (3)	0.65268 (8)	0.0284 (4)	
O2	0.49925 (5)	1.3547 (3)	0.68019 (8)	0.0235 (4)	
O3	0.17182 (6)	0.9470 (4)	0.39349 (9)	0.0400 (5)	
H3A	0.1397	0.9621	0.3761	0.060*	
O4	0.14358 (6)	0.6002 (4)	0.32174 (10)	0.0418 (5)	
O1W	0.45497 (6)	1.8957 (3)	0.75397 (8)	0.0285 (4)	
H1A	0.4237	1.9018	0.7291	0.043*	
H1B	0.4664	2.0419	0.7755	0.043*	

### Atomic displacement parameters (Å^2^)

**Table:**

	*U*^11^	*U*^22^	*U*^33^	*U*^12^	*U*^13^	*U*^23^
Zn1	0.0264 (2)	0.0185 (2)	0.0267 (2)	0.000	0.01250 (18)	0.000
C1	0.0207 (12)	0.0198 (12)	0.0214 (11)	0.0020 (9)	0.0097 (9)	0.0046 (9)
C2	0.0213 (12)	0.0211 (12)	0.0170 (10)	0.0001 (9)	0.0072 (9)	0.0013 (9)
C3	0.0182 (11)	0.0258 (13)	0.0203 (11)	0.0023 (9)	0.0091 (9)	−0.0006 (9)
C4	0.0192 (11)	0.0231 (12)	0.0203 (11)	0.0007 (10)	0.0085 (9)	0.0003 (9)
C5	0.0199 (12)	0.0260 (13)	0.0200 (11)	−0.0011 (10)	0.0085 (9)	−0.0025 (10)
C6	0.0234 (12)	0.0278 (13)	0.0249 (12)	0.0009 (10)	0.0118 (10)	−0.0037 (10)
C7	0.0193 (12)	0.0304 (14)	0.0275 (12)	0.0006 (10)	0.0095 (10)	−0.0024 (10)
C8	0.0241 (13)	0.0347 (15)	0.0333 (13)	−0.0041 (12)	0.0087 (11)	−0.0099 (12)
C9	0.0267 (13)	0.0325 (15)	0.0316 (13)	0.0008 (11)	0.0112 (11)	−0.0095 (11)
C10	0.0254 (13)	0.0258 (13)	0.0231 (12)	0.0021 (10)	0.0111 (10)	−0.0015 (9)
C11	0.0224 (12)	0.0214 (12)	0.0199 (11)	0.0032 (10)	0.0092 (9)	0.0031 (9)
C12	0.0224 (12)	0.0303 (13)	0.0240 (11)	0.0052 (10)	0.0115 (10)	−0.0001 (10)
C13	0.0175 (11)	0.0293 (13)	0.0233 (11)	0.0010 (10)	0.0072 (9)	0.0016 (10)
C14	0.0243 (13)	0.0325 (14)	0.0298 (13)	0.0010 (11)	0.0115 (11)	−0.0028 (11)
C15	0.0287 (13)	0.0251 (13)	0.0299 (12)	0.0058 (11)	0.0140 (11)	−0.0020 (10)
C16	0.0380 (16)	0.0498 (18)	0.0353 (14)	0.0066 (14)	0.0236 (13)	−0.0029 (13)
C17	0.053 (2)	0.063 (2)	0.0472 (17)	0.0068 (16)	0.0314 (16)	0.0143 (16)
N1	0.0209 (10)	0.0261 (11)	0.0233 (10)	0.0026 (8)	0.0100 (8)	−0.0044 (8)
O1	0.0211 (9)	0.0290 (9)	0.0296 (9)	0.0026 (7)	0.0083 (7)	−0.0079 (7)
O2	0.0150 (8)	0.0241 (9)	0.0250 (8)	−0.0015 (7)	0.0047 (7)	−0.0010 (7)
O3	0.0206 (9)	0.0479 (12)	0.0461 (11)	0.0008 (8)	0.0122 (8)	−0.0178 (9)
O4	0.0200 (9)	0.0454 (12)	0.0514 (11)	−0.0048 (8)	0.0109 (8)	−0.0197 (9)
O1W	0.0164 (8)	0.0239 (9)	0.0367 (9)	−0.0017 (7)	0.0065 (7)	−0.0062 (7)

### Geometric parameters (Å, º)

**Table:**

Zn1—O1	2.4040 (15)	C9—H9	0.9500
Zn1—O2	2.0392 (15)	C10—N1	1.381 (3)
Zn1—O1W	1.9824 (16)	C11—N1	1.385 (3)
C1—O1	1.252 (3)	C11—C12	1.389 (3)
C1—O2	1.281 (3)	C12—C13	1.379 (3)
C1—C2	1.484 (3)	C12—H12	0.9500
C2—C3	1.387 (3)	C13—H13	0.9500
C2—C13	1.409 (3)	C14—O4	1.216 (3)
C3—C4	1.389 (3)	C14—O3	1.313 (3)
C3—H3	0.9500	C15—N1	1.448 (3)
C4—C11	1.414 (3)	C15—C16	1.475 (3)
C4—C5	1.452 (3)	C15—H15A	0.9900
C5—C6	1.387 (3)	C15—H15B	0.9900
C5—C10	1.408 (3)	C16—C17	1.311 (4)
C6—C7	1.388 (3)	C16—H16	0.9500
C6—H6	0.9500	C17—H17A	0.9500
C7—C8	1.410 (3)	C17—H17B	0.9500
C7—C14	1.475 (3)	O3—H3A	0.8721
C8—C9	1.371 (3)	O1W—H1A	0.8438
C8—H8	0.9500	O1W—H1B	0.8534
C9—C10	1.401 (3)		
			
O1W—Zn1—O1W^i^	92.67 (10)	C5—C6—H6	120.6
O1W—Zn1—O2	138.07 (6)	C7—C6—H6	120.6
O1W^i^—Zn1—O2	100.30 (6)	C6—C7—C8	120.3 (2)
O1W—Zn1—O2^i^	100.30 (6)	C6—C7—C14	121.8 (2)
O1W^i^—Zn1—O2^i^	138.07 (6)	C8—C7—C14	117.9 (2)
O2—Zn1—O2^i^	96.14 (9)	C9—C8—C7	122.2 (2)
O1W—Zn1—O1^i^	126.12 (6)	C9—C8—H8	118.9
O1W^i^—Zn1—O1^i^	81.91 (6)	C7—C8—H8	118.9
O2—Zn1—O1^i^	95.28 (6)	C8—C9—C10	116.9 (2)
O2^i^—Zn1—O1^i^	58.24 (6)	C8—C9—H9	121.5
O1W—Zn1—O1	81.91 (6)	C10—C9—H9	121.5
O1W^i^—Zn1—O1	126.12 (6)	N1—C10—C9	128.6 (2)
O2—Zn1—O1	58.24 (6)	N1—C10—C5	109.44 (19)
O2^i^—Zn1—O1	95.28 (6)	C9—C10—C5	121.9 (2)
O1^i^—Zn1—O1	142.07 (8)	N1—C11—C12	129.1 (2)
O1W—Zn1—C1^i^	117.02 (7)	N1—C11—C4	108.95 (19)
O1W^i^—Zn1—C1^i^	110.17 (7)	C12—C11—C4	121.9 (2)
O2—Zn1—C1^i^	95.62 (6)	C13—C12—C11	117.5 (2)
O2^i^—Zn1—C1^i^	29.40 (6)	C13—C12—H12	121.3
O1^i^—Zn1—C1^i^	28.87 (6)	C11—C12—H12	121.3
O1—Zn1—C1^i^	119.98 (7)	C12—C13—C2	121.7 (2)
O1W—Zn1—C1	110.17 (7)	C12—C13—H13	119.1
O1W^i^—Zn1—C1	117.01 (7)	C2—C13—H13	119.1
O2—Zn1—C1	29.39 (6)	O4—C14—O3	123.2 (2)
O2^i^—Zn1—C1	95.62 (7)	O4—C14—C7	122.3 (2)
O1^i^—Zn1—C1	119.98 (6)	O3—C14—C7	114.5 (2)
O1—Zn1—C1	28.87 (6)	N1—C15—C16	114.6 (2)
C1^i^—Zn1—C1	109.29 (10)	N1—C15—H15A	108.6
O1—C1—O2	119.3 (2)	C16—C15—H15A	108.6
O1—C1—C2	121.8 (2)	N1—C15—H15B	108.6
O2—C1—C2	118.8 (2)	C16—C15—H15B	108.6
O1—C1—Zn1	67.95 (12)	H15A—C15—H15B	107.6
O2—C1—Zn1	51.41 (10)	C17—C16—C15	126.1 (3)
C2—C1—Zn1	169.20 (16)	C17—C16—H16	116.9
C3—C2—C13	120.1 (2)	C15—C16—H16	116.9
C3—C2—C1	120.5 (2)	C16—C17—H17A	120.0
C13—C2—C1	119.4 (2)	C16—C17—H17B	120.0
C2—C3—C4	119.3 (2)	H17A—C17—H17B	120.0
C2—C3—H3	120.4	C10—N1—C11	108.79 (18)
C4—C3—H3	120.4	C10—N1—C15	125.86 (19)
C3—C4—C11	119.4 (2)	C11—N1—C15	125.34 (19)
C3—C4—C5	134.1 (2)	C1—O1—Zn1	83.19 (13)
C11—C4—C5	106.49 (19)	C1—O2—Zn1	99.20 (13)
C6—C5—C10	119.9 (2)	C14—O3—H3A	105.3
C6—C5—C4	133.7 (2)	Zn1—O1W—H1A	126.9
C10—C5—C4	106.33 (19)	Zn1—O1W—H1B	120.8
C5—C6—C7	118.7 (2)	H1A—O1W—H1B	110.9

Symmetry code: (i) −*x*+1, *y*, −*z*+3/2.

### Hydrogen-bond geometry (Å, º)

**Table:**

*D*—H···*A*	*D*—H	H···*A*	*D*···*A*	*D*—H···*A*
O1*W*—H1*A*···O4^ii^	0.84	1.81	2.654 (2)	177
O1*W*—H1*B*···O2^iii^	0.85	1.88	2.728 (2)	170
O3—H3*A*···O1^ii^	0.87	1.77	2.634 (2)	173

Symmetry codes: (ii) −*x*+1/2, −*y*+5/2, −*z*+1; (iii) −*x*+1, *y*+1, −*z*+3/2.
